# Categorizing Vaccine Confidence With a Transformer-Based Machine Learning Model: Analysis of Nuances of Vaccine Sentiment in Twitter Discourse

**DOI:** 10.2196/29584

**Published:** 2021-10-08

**Authors:** Per E Kummervold, Sam Martin, Sara Dada, Eliz Kilich, Chermain Denny, Pauline Paterson, Heidi J Larson

**Affiliations:** 1 Vaccine Research Department FISABIO-Public Health Valencia Spain; 2 Centre for Clinical Vaccinology and Tropical Medicine University of Oxford Oxford United Kingdom; 3 Rapid Research Evaluation and Appraisal Lab Departament of Targeted Intervention University College London London United Kingdom; 4 Faculty of Epidemiology and Population Health London School of Hygiene & Tropical Medicine London United Kingdom; 5 Ethox Centre, Nuffield Department of Population Health Big Data Institute University of Oxford Oxford United Kingdom; 6 UCD Centre for Interdisciplinary Research, Education and Innovation in Health Systems School of Nursing, Midwifery and Health Systems University College Dublin Dublin Ireland; 7 Faculty of Science Vrije Universiteit Amsterdam Amsterdam Netherlands; 8 NIHR Health Protection Research Unit London United Kingdom; 9 Institute of Health Metrics and Evaluation University of Washington Seattle, WA United States; 10 Chatham House Centre on Global Health Security The Royal Institute of International Affairs London United Kingdom

**Keywords:** computer science, information technology, public health, health humanities, vaccines, machine learning

## Abstract

**Background:**

Social media has become an established platform for individuals to discuss and debate various subjects, including vaccination. With growing conversations on the web and less than desired maternal vaccination uptake rates, these conversations could provide useful insights to inform future interventions. However, owing to the volume of web-based posts, manual annotation and analysis are difficult and time consuming. Automated processes for this type of analysis, such as natural language processing, have faced challenges in extracting complex stances such as attitudes toward vaccination from large amounts of text.

**Objective:**

The aim of this study is to build upon recent advances in transposer-based machine learning methods and test whether transformer-based machine learning could be used as a tool to assess the stance expressed in social media posts toward vaccination during pregnancy.

**Methods:**

A total of 16,604 tweets posted between November 1, 2018, and April 30, 2019, were selected using keyword searches related to maternal vaccination. After excluding irrelevant tweets, the remaining tweets were coded by 3 individual researchers into the categories *Promotional*, *Discouraging*, *Ambiguous*, and *Neutral or No Stance*. After creating a final data set of 2722 unique tweets, multiple machine learning techniques were trained on a part of this data set and then tested and compared with the human annotators.

**Results:**

We found the accuracy of the machine learning techniques to be 81.8% (*F* score=0.78) compared with the agreed score among the 3 annotators. For comparison, the accuracies of the individual annotators compared with the final score were 83.3%, 77.9%, and 77.5%.

**Conclusions:**

This study demonstrates that we are able to achieve close to the same accuracy in categorizing tweets using our machine learning models as could be expected from a single human coder. The potential to use this automated process, which is reliable and accurate, could free valuable time and resources for conducting this analysis, in addition to informing potentially effective and necessary interventions.

## Introduction

### Background

Although individuals have been found to share different thoughts, questions, and concerns about vaccines on social media [1], studies of the vaccine discourse on social media [2] indicate that concerns, and indeed the sharing of misinformation in particular, are amplified [3]. What is of concern is the number of imprecise and inaccurate articles available with regard to vaccinations.

Multiple studies have been conducted to monitor vaccination discussions on social media [4-6]. Addressing misunderstandings and inaccuracies as early as possible is vital to making sound vaccine policies. However, there is currently insufficient research on how to effectively categorize the nuances in the perceptions of sentiment toward vaccines in the large volume of vaccine data shared daily on social media. Being able to monitor and understand the spread and traction of misinformation in social media on a larger global scale is key to mitigating the negative impact of such information.

Although the data retrieved from social and news media might not be representative of the entire population, they provide a snapshot of discussions and thoughts, and the trends observed here are still thought to be of vital importance to understanding emerging issues of concern as well as the link between misinformation on news and social media platforms and the effect of this misinformation on vaccination confidence and uptake. To detect such trends, however, we need an in-depth understanding of the content of these messages. Although qualitative methods might provide this insight, the sheer volume of news and social media content makes it difficult to apply these methods to conversations among entire populations over time. Machine learning and natural language processing (NLP) have the potential to handle huge amounts of information. However, concerns over accuracy, especially when dealing with the complexity of the language used to express opinions about vaccines, have prevented these methods from being very effective.

Sentiment analysis in machine learning refers to the process of automatically determining whether the author of a piece of text is in favor of, against, or neutral toward the subject of the statement. This is slightly different from stance detection, which involves automatically determining the author’s attitude toward a proposition or target [7]. Although sentiment analysis can look only at the tone of a particular statement, stance detection often refers to a target outside of the particular statement.

The author of a tweet could express a positive attitude toward vaccination by expressing negativity toward people opposing vaccines (for instance, so-called *antivaxxers*). This double negation would then be interpreted as *positive* or *promotional*. This could be referred to as the *author’s sentiment toward vaccination*, but because *sentiment* is often used for referring to the *sentiment of the statement*, we find it less confusing to refer to this as the *author’s stance* toward vaccination. This distinction is particularly important when studying an issue as complex as vaccination because many texts often express strong opinions about vaccination without addressing vaccines directly. The distinction can be illustrated using the examples presented in Table 1.

Historically, NLP has often focused on ordinary sentiment analysis. This is technically a much easier task, but it is less useful from a sociological point of view. In contrast to *sentiment*, a person’s *stance* toward a target can be expressed using either negative or positive language. People could, for instance, switch from opposing *abortion* to promoting *prolife* without changing their basic stance. In a sociological analysis, we would usually be more interested in the stance that people have toward a topic or target than the sentiment expressed in a particular statement.

**Table 1:** Difference between sentiment and stance.

Text	Sentiment (subject)	Stance (target)
Vaccines save lives	Positive (vaccines)	Positive (vaccines)
Antivaxxers kill people with their misinformation	Negative (antivaxxers)	Positive (vaccines)
Trust your doctor’s knowledge regarding vaccines	Positive (physician’s knowledge)	Positive (vaccines)
Antivaxxers tell the real truth about vaccines	Positive (antivaxxers)	Negative (vaccines)

### Objective

The aim of this study is to look more deeply into attitudes toward maternal vaccination and how well the task of detecting stance in tweets can be accomplished by using multiple machine learning methods. We attempt to quantify how accurately such tweets can be categorized by trained annotators and how this compares with newer machine learning methods.

## Methods

### Overview

This research collected 16,605 Twitter messages (tweets) published over 6 months between November 1, 2018, and April 30, 2019, from Meltwater [8], a media intelligence system. This data set was collected and coded to complement a larger research study on sentiments and experiences around maternal vaccination across 15 countries (Australia, Brazil, Canada, France, Germany, India, Italy, Mexico, Panama, South Africa, South Korea, Spain, Taiwan, the United Kingdom, and the United States). Non-English tweets were translated into English using Google Translate script (Alphabet Inc). Multimedia Appendix 1 includes the search queries used in this study. Before annotating, all usernames and links were replaced by a common tag. This served two purposes: it preserved anonymity, and it limited potential bias based on the coder’s interpretation of the username. The target of the analysis should be to decipher what the text is actually telling the reader about the writer’s stance toward vaccination.

In this study, *maternal vaccination* typically refers to the vaccines that are recommended by health authorities for pregnant women.

Individual tweets were manually coded into stance categories (Textbox 1). Stance was categorized across four sentiments toward maternal vaccines: *Promotional* (in favor of maternal vaccines), *Ambiguous* (uncertainty with mixed sentiment toward maternal vaccines), *Discouraging* (against maternal vaccines), and *No stance* (statements or facts about maternal vaccines that do not reveal the author’s stance). Although it can be argued that some of the categories can be ordered, we treated them as nominal variables, not ordinal variables, in the analysis. Therefore, a tweet stating that pregnant women should take the tetanus vaccine but not the measles vaccine is considered a promotional post in favor of maternal vaccines because it encourages following the current health recommendations.

**Stance categorized across four sentiments toward maternal vaccines.**

**Stance categories and their definitions**

- Promotional
  Posts communicate public health benefits or safety of maternal vaccination.
  Posts contain positive tones, supportive or encouraging toward maternal vaccination.
- Ambiguous
  Posts contain indecision and uncertainty on the risks or benefits of maternal vaccination, or they are ambiguous.
  Posts contain disapproving and approving information.
  Posts describe risks of not vaccinating during pregnancy.
  Posts refute claims that maternal vaccines are dangerous.
- Discouraging
  Posts contain negative attitudes toward, or arguments against maternal vaccines.
  Posts contain questions regarding effectiveness or safety or possibility of adverse reactions (eg, links to disability or autism).
  Posts discourage the use of recommended maternal vaccines.
- Neutral or no stance
  Posts contain no elements of uncertainty or promotional or negative content. These are often not sentiments expressed on the web but rather statements that are devoid of emotion. This category includes factual posts pointing to articles on maternal vaccines (eg, Study on effectiveness of maternal flu vaccine).

### Cleaning the Data Set

After the initial annotating, the data set was cleaned for duplicates and semiduplicates. Semiduplicates are tweets in which a few characters differ but the meaning is unchanged. A typical example is a retweeted post with the *RT:* prefix. Another example is a tweet suffixed (by a user or bot) with a few random characters to avoid being recognized (by Twitter detection algorithms) as a mass posting. To detect semiduplicates, we used a nonnormalized Levenshtein distance of less than 30 for tweets with more than 130 characters. For shorter tweets, the distance was scaled. The validity of the deduplication algorithm was qualitatively evaluated by the annotators. We were aiming for a *greedy* algorithm that identified too many semiduplicates rather than too few. Although this could slightly affect the size of the training set, it was considered to be of greater importance to prevent tweets that looked too similar from being included in both the training and test data sets. We have open sourced the Python code that we developed for cleaning and removing duplicates and made it available in our web-based GitHub repository [9].

As the stance of the tweet should be determined solely based on the content of the text, we deidentified usernames and URLs. Apart from cleaning usernames and URLs, the aim was to ensure that the input to the machine learning algorithm was exactly the same as that presented to the human annotators. In this respect, this served to both preserve anonymity and limit potential bias arising from the annotator’s interpretation of the usernames. This also helped fulfill ethical responsibilities as well as the European Union’s General Data Protection Regulation guidelines. In all, 3 independent annotators screened all posts for inclusion, excluding posts that were not about the vaccines administered during pregnancy. The annotators also met to agree on the final posts included in the analysis [10].

**Figure 1:**
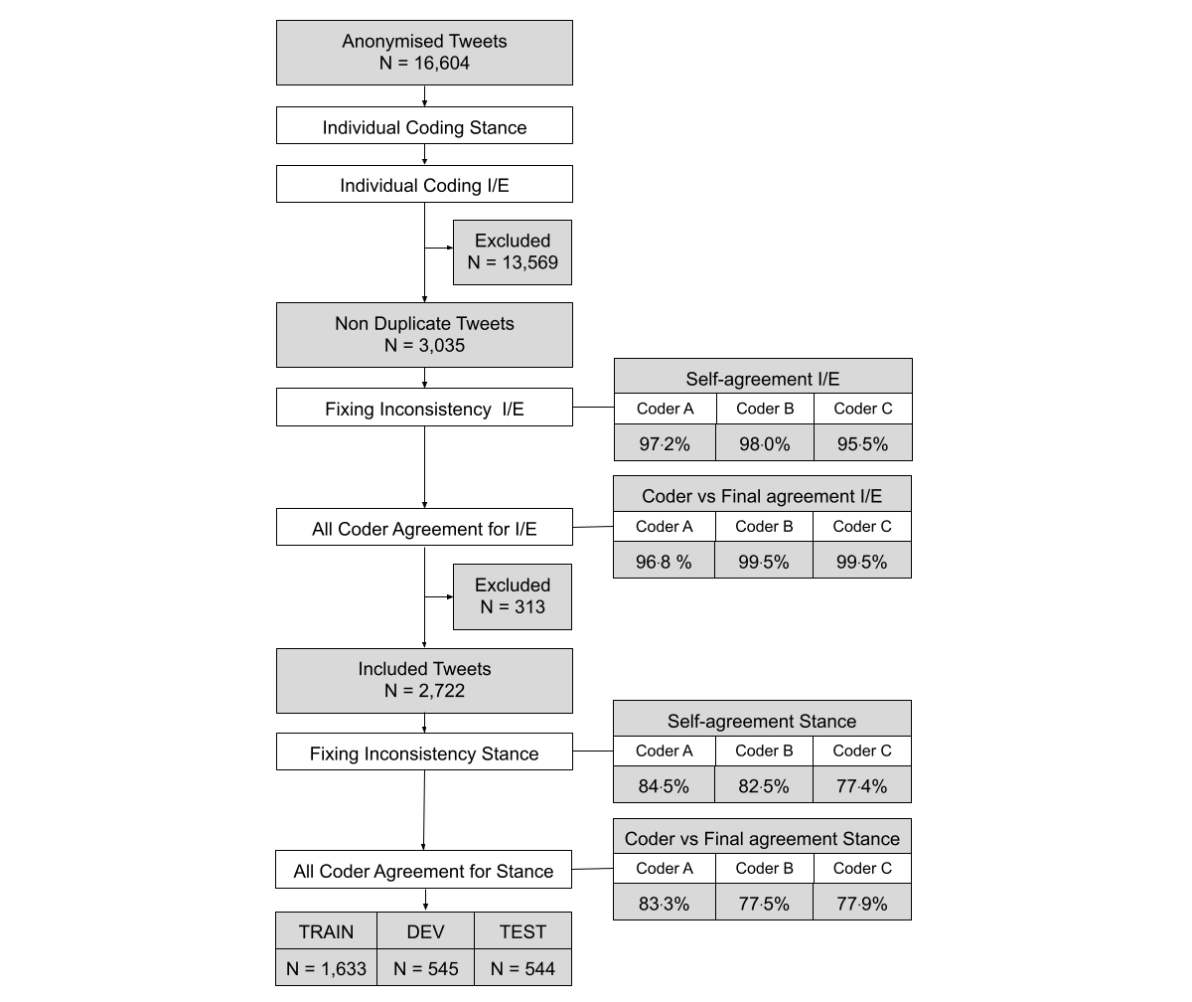
Screening and annotating procedure. DEV: development data set; I/E: include or exclude.

Deduplication was conducted after the first round of annotating, and the annotators were then asked to recode any tweet for which they had provided inconsistent annotating. For example, there were instances where the same coder coded identical tweets inconsistently. From the tweets that appeared only twice in the material, we calculated a self-agreement score both for include or exclude and for stance. This was done to illustrate some of the potential challenges of manual annotating (Figure 1).

### Bidirectional Encoder Representations From Transformers

The main model was based on the newest (May 2019) Whole Word Masking variant of Google’s Bidirectional Encoder Representations from Transformers (BERT) [11]. When published in late 2018, the model demonstrated state-of-the-art results on 11 NLP tasks, including the Stanford Question Answering Dataset version 1.1 (The Stanford Natural Language Processing Group) [12]. BERT is a bidirectional, contextual encoder built on a network architecture called Transformer, which is based solely on attention mechanisms [13]. The main part of the training can be performed on unsupervised and unlabeled text corpora such as Wikipedia, and the pretrained weights [14] are trained solely on this general corpus.

We trained the model on a domain-specific corpus to expose the model to the vocabulary that is typically used in vaccination posts. We started creating domain-specific pretraining data by downloading 5.9 million tweets acquired by keyword searches related to vaccine and vaccination (Multimedia Appendix 2). The set was downloaded from Meltwater and preprocessed in the same way as the maternal vaccine tweets (ie, deduplication and username or link anonymization). The BERT architecture depends on carrying out unsupervised pretraining using a technique called Masked Language Modeling and Next Sentence Prediction. The latter method requires each text segment to have at least two sentences. Therefore, we filtered out all tweets that did not satisfy this criterion, reducing the data set from 1.6 million to 1.1 million tweets. A later study by Liu et al [15] pointed out that the Next Sentence Prediction task is not as effective as expected and that it might be beneficial to train the model only on the Masked Language Modeling task. As we have such a large number of short texts, this would have extended our data set. We refer to this data set as the vaccine-tweet data set.

**Figure 2:**
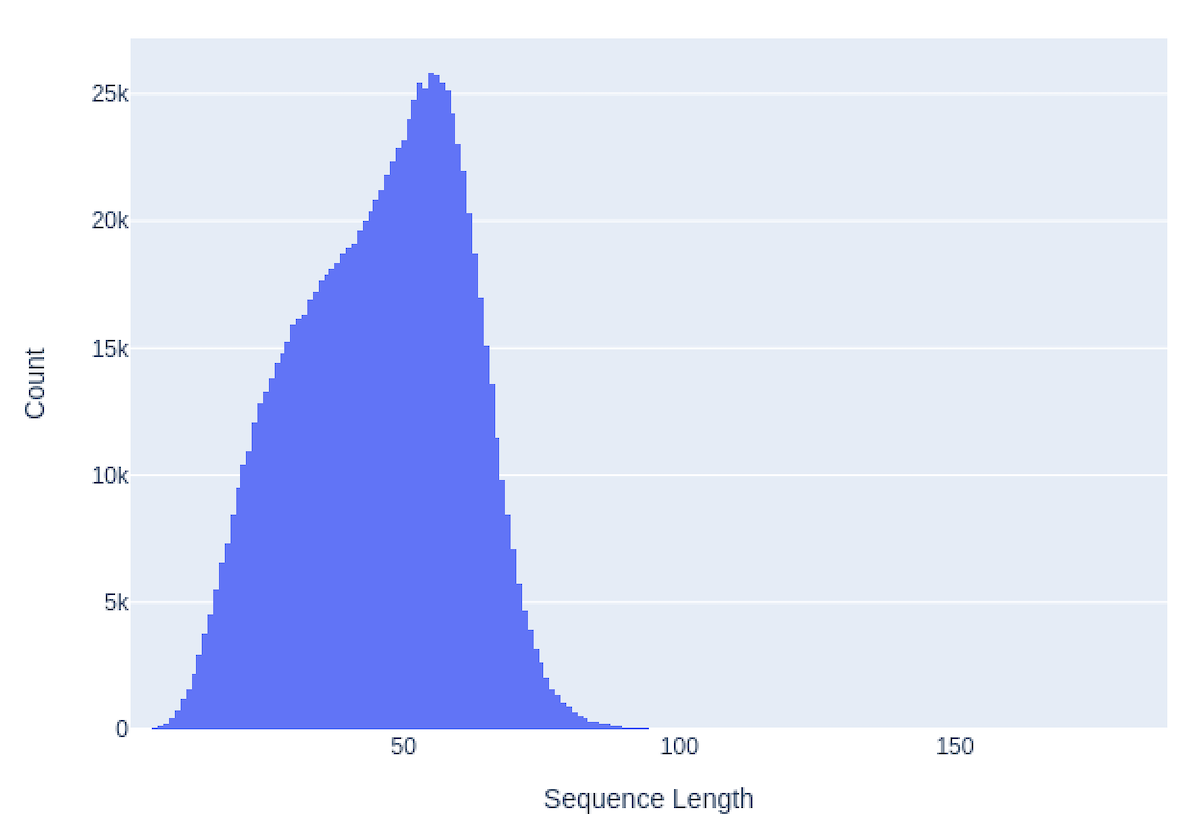
Number of tokens in each tweet (count per million tweets).

We tokenized the tweets using the BERT vocabulary and limited the sequence length to 96 tokens. By limiting the sequence length, we were able to increase the batch size, which is known to have a positive effect on performance. Figure 2 shows the sequence length of the downloaded tweets, showing that this trimming would affect less than one in thousand tweets. Tweets longer than 96 tokens were manually examined, confirming that these were mainly repetitive sequences and that the trimming did not affect the meaning (eg, a statement followed by strings of varying lengths of repeated characters such as *......* or *????*). We have further addressed discourse distribution, labeling, and word balance in the word corpus in the study by Martin et al [10].

In addition, we acquired a data set with a total of 201,133 vaccine-related news articles from the Vaccine Confidence Project (London School of Hygiene & Tropical Medicine) media archive. The articles were collected by automated keyword searches from several sources, including Google News, HealthMap, and Meltwater. It is an extensive collection of vaccine-related articles in English from both news media and blogs. The search criteria have been developed over the years, which is why they have varied slightly, but they are very similar to the list presented in Multimedia Appendix 2. We refer to this data set as the vaccine news data set. We chose not to pretrain the model on a maternal vaccine–specific data set because we wanted the encoder representations to also be used on other vaccine-related topics. All pretraining was carried out using a learning rate of 2e-5, a batch size of 216, and a maximum sequence length of 96.

These domain-specific pretrained weights were the starting points for the classification of the maternal vaccination tweets. The manually classified maternal vaccination tweets were preprocessed in the same way as the tweets in the vaccine-tweet data set and then divided into training, development, and test data sets in the ratio 60:20:20 (N=1633:545:544).

### Fine-tuning

**Figure 3:**
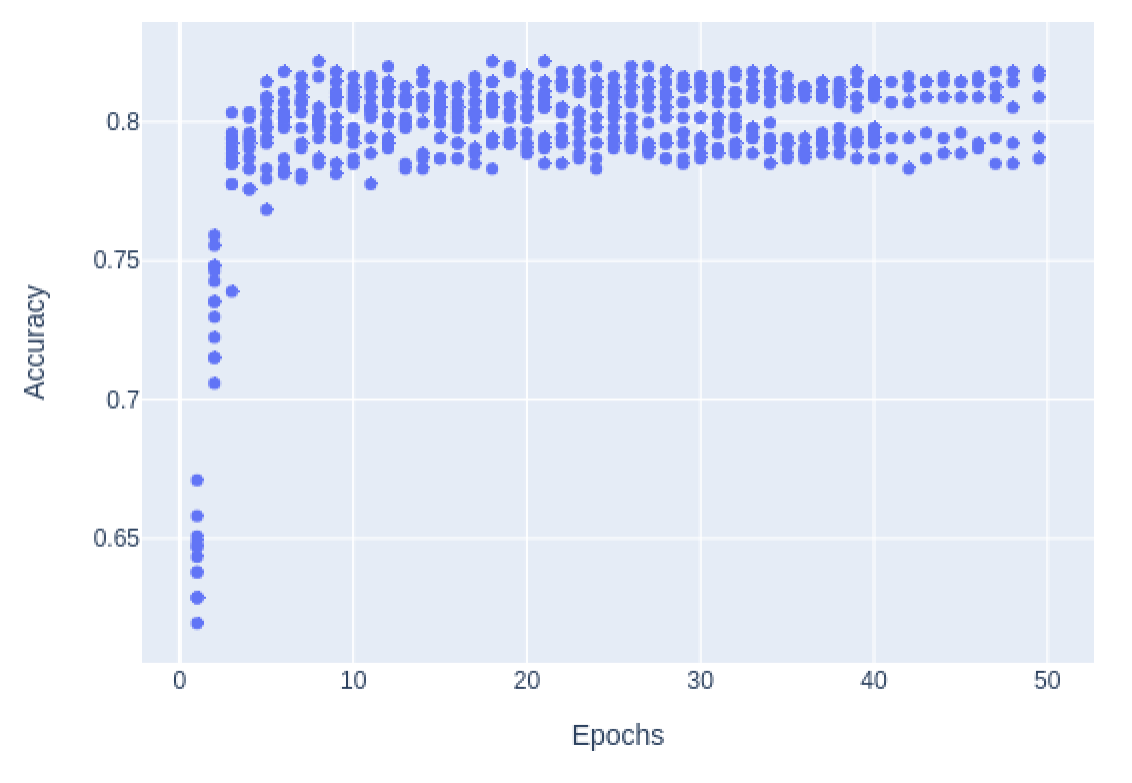
Data evaluation of pretraining accuracy.

The pretraining of transposer models is a very slow process, but when these pretrained weights are determined, the final fine-tuning step is fast. To our knowledge, the best way of comparing the various pretrained models is by comparing their performances after fine-tuning. Figure 3 shows that the fine-tuning did not improve performance after 15 epochs but that there was considerable variance among the runs. For this reason, all pretrained models were evaluated using an average of 10 fine-tuned runs each at 15 epochs.

To obtain a baseline score for comparative machine learning models, various traditional and well-established networks were trained. The aim was to use well-established networks with known performance against standardized data sets for sentiment and stance analysis. The benchmark architectures, the neural network, and the long short-term memory (LSTM) networks with and without Global Vectors for Word Representation (GloVe; The Stanford Natural Language Processing Group) word embeddings were all taken from *Deep Learning With Python* by Chollet [16].

To verify that the neural network was able to solve other neural network tasks, we tested the network structures on one of the most basic NLP tasks: predicting positive and negative sentiments in IMDb (Amazon, Inc) movie reviews [17].

The final domain-specific pretraining and fine-tuning were carried out on a Cloud TPU (Tensor Processing Unit) v2-8 node with 8 cores and 64 gibibytes memory and an estimated performance of 180 teraflops (Google Cloud). Domain-specific pretraining was carried out for 2 weeks, but, as shown in Figure 3, there were no measurable improvements after a few days of running. Fine-tuning requires fewer computing resources and is usually completed in a few minutes on this platform.

## Results

### Overview

In total, 3 annotators individually coded 2722 tweets. Of these 2722 tweets, 1559 (57.27%) were coded identically, with a Fleiss agreement score of κ=0.56. After meeting and discussing the tweets that they disagreed on, the annotators agreed on the annotating of all the remaining tweets. Although the annotators agreed on a final category for every tweet, they also reported that 6.83% (186/2722) of tweets “could be open to interpretation.” Comparing the final agreed annotating after the discussions with the annotators’ initial annotating, the accuracies of the individual annotators were 83.3%, 77.9%, and 77.5%. The accuracy of the machine learning model was also calculated with regard to the final agreed annotating.

One of the basic neural networks for NLP consists of two fully connected layers. For our data set, this only provided an accuracy of 43.7%. Thus, the network was not able to obtain a better result than simply predicting the overrepresented task *Promotional* for all data points. Adding pretrained GloVe word embeddings to this structure resulted in a slightly better performance, with a maximum accuracy of 55.5% on the test data set. However, both approaches overtrained after just a few epochs with data sets of this size.

To evaluate the reason for this low accuracy, we tested the same network on the IMDb data set, setting the same number of training examples (N=1633). In this case, the network achieved an accuracy of more than 80% even without the GloVe word embeddings, showing that the low accuracy was related to the difficulty of the maternal vaccine categorization.

The modern LSTM model is a recurrent neural network with a memory module. This model architecture was considered state-of-the-art a couple of years ago. We were able to obtain an accuracy of 63.1% here and can improve this to 65.5% by adding pretrained GloVe word embeddings.

### Main Research Target

Our main research target was to investigate whether state-of-the-art NLP models could be improved by using the BERT architecture. Using the original pretrained weights, we achieved an average accuracy of 76.7% when fine-tuning for 15 epochs.

Starting from the original weights, the model weights were pretrained on the larger vaccine news data set for 1 million epochs. At various checkpoints (0 E, 250,000 E, 500,000 E, 750,000 E, and 1,000,000 E), the model was forked and then trained on the smaller and more specific vaccine-tweet data set.

**Figure 4:**
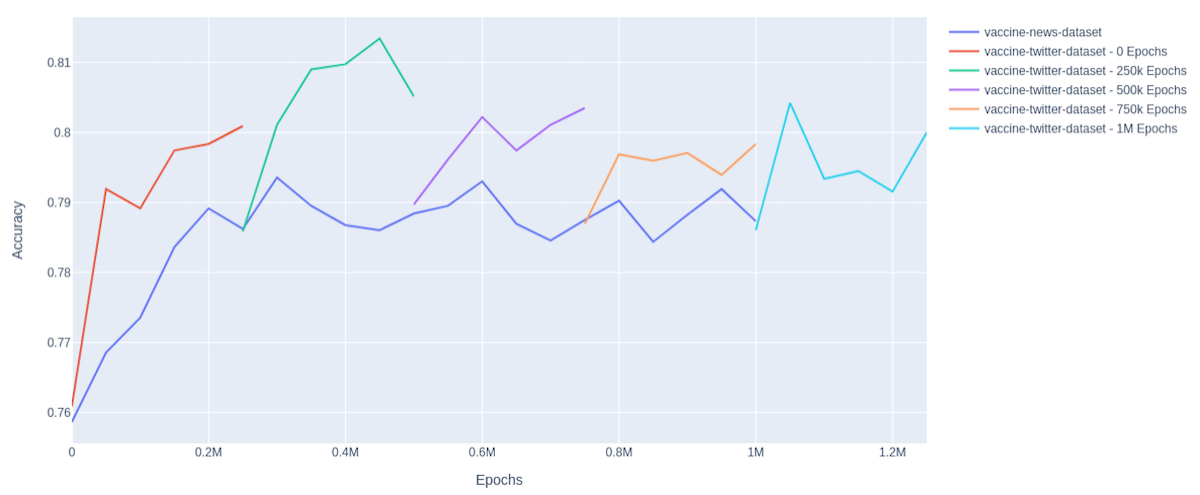
Average of pretraining accuracy.

At each of the checkpoints, the network was fine-tuned on the manually labeled tweets for 15 epochs, and the average of 10 runs is shown in Figure 4. Using pretraining on domain-specific content, the accuracy reached a peak of approximately 79% when training only on the vaccine news data set. However, by training first on the vaccine news data set for 250,000 epochs and then on the vaccine news data set for an additional 200,000 epochs, we were able to obtain an accuracy of 81.8%. For a comparison of accuracies, see Table 2.

**Table 2:** Accuracy comparisons.

			Accuracy		*F* score
**Coder average**			0.795861		0.739396
	Coder 1: EK	0.833211		0.796272	
	Coder 2: SCM	0.775165		0.710356	
	Coder 3: SD and CD	0.779206		0.711559	
Neural network: no embeddings			0.436697		0.436697
Neural network: GloVe^a^ word embeddings			0.544954		0.457813
LSTM^b^: no embeddings			0.631193		0.549997
LSTM+GloVe word embeddings			0.655046		0.593942
BERT^c^: default weights			0.766972^d^		0.718878
BERT: domain-specific			0.818349^d^		0.775830

^a^GloVe: Global Vectors for Word Representation.

^b^LSTM: long short-term memory.

^c^BERT: Bidirectional Encoder Representations from Transformers.

^d^The final accuracy scores for the Bidirectional Encoder Representations from Transformers–based models are based on selecting the best network from the results based on the results from the development data set. The reported numbers are from evaluating the training data set.

## Discussion

### Principal Findings

The categories chosen in this study underwent several revisions to ensure that they could be clearly understood. The annotators were fluent English speakers with a postgraduate degree and several years of work experience in the field.

Some of the nuances contained in the tweets meant that it was difficult to categorize them as definitively one stance. Thus, even when the same coder was asked to code a nearly identical tweet at a later time, they chose the same code four out of five times. After being given a second opportunity to code all duplicate tweets that had inconsistencies, the annotators met and discussed the categories that they disagreed on. The average final accuracy was then 79.6%.

Ideally, the correct annotating should be the annotating that an average of a large number of experienced annotators would have chosen. Limiting the number of annotators to 3 resulted in cases where all annotators by chance coded the same tweet identically and erroneously and cases where none of the annotators chose the categories that a larger number of annotators would have chosen. It is therefore reasonable to assume that the accuracy of 79.6% might be slightly optimistic in terms of what can be expected from an average human coder, even one who has long experience in the area.

The task of annotating is challenging because it is open for interpretation, which is a challenge that NLP also struggles with. Our tests showed that a simple neural network that had no problem achieving an accuracy of more than 80% on an IMDb movie review task was unable to predict anything better than the most prevalent category when it was tested on maternal vaccination tweets.

Unsurprisingly, LSTM networks perform better than ordinary neural networks. Pretrained embeddings help in all cases. Using GloVe word embeddings increases the accuracy. With the LSTM network, we achieved an accuracy of 63.1% and improved this to 65.5% by adding pretrained GloVe word embeddings. However, LSTM networks still lag behind what could be considered human accuracy.

In contrast, transformer-based architectures perform significantly better. By using the pretrained openly available BERT weights, we achieved an accuracy of 76.7%. This is approximately the same level of accuracy achieved by the coder with the lowest accuracy in this study.

Domain-specific pretraining also shows potential. Although pretraining does require some computing power, it does not require manual annotating, which could entail high costs in terms of time and resources. It is also worth noting that we deliberately trained the model only on general vaccine terms. We did not perform optimizations specifically for the domain of maternal vaccines. The main reason for this is that we wanted weights that were transferable to other tasks in the field of vaccines.

In our setting, the best result of 81.8% accuracy was achieved after initial training on news articles about vaccines and then training on vaccine-related tweets. This accuracy is better than the average of the 3 annotators, even after the annotators had carried out multiple annotatings of the same tweet and had been given the opportunity to recode any inconsistencies.

In our opinion, it is doubtful that any individual coder would achieve more than 90% accuracy on this task simply because it is difficult, even with a much larger number of annotators, to agree on an absolute categorization. There will always be tweets that are open to interpretation, preventing a hard target of absolute accuracy.

### Limitations

We used a limited data set, especially for the tweet data set containing only 1 million vaccine-related tweets. It is also reasonable to assume that pretraining on a larger data set of non–vaccine-specific tweets could have a positive effect because the language of tweets is quite different from that of other texts. Enlarging the data sets is an easy way of potentially increasing the accuracy.

Although they are accurate, transformer-based models are demanding to run to analyze large amounts of text. This could be a challenge when used for monitoring purposes. However, most likely, this is a problem that will lessen in the future.

After Google released BERT late in 2018, there have been multiple general improvements made by Facebook, Microsoft, and Google to the model’s transformer-based architecture to improve the base models [15,18,19]. These have not been implemented in this study. There is currently significant research activity in the field, and it is reasonable to assume that implementing these improvements in the base model and restarting the domain-specific pretraining checkpoints would lead to higher accuracy in our categorization.

### Conclusions

Being able to categorize and understand the overall stance in social media conversations about vaccinations, especially in terms of identifying clusters of discouraging or ambiguous conversations, will make it easier to spot activities that may signal vaccine hesitancy or a decline in vaccine confidence with greater speed and more accuracy. To manually, and continually, monitor these conversations in today’s information society is near impossible. In that respect, it has always been obvious that NLP has huge potential because it can process an enormous amount of textual information.

However, so far, NLP has only been able to solve very easy tasks and is unable to handle the nuances in language related to complicated issues (eg, attitudes toward vaccination). The new advances in transformer-based models indicate that they are about to become a useful tool in this area, opening up a new area for social research.

We have demonstrated that with a training data set of approximately 1600 tweets, we were able to obtain at least the accuracy that should be expected of a trained human coder in categorizing the stance of maternal vaccination discussions on social media. Although there are benefits to increasing this accuracy even more, the main research challenge is to reduce the number of training samples. So far, this has been an underprioritized area of research and an area where we should expect advances in the future. The real benefit from the technology will first be apparent when we are able to do this kind of categorization with only a few initial examples. Being able to categorize text in large corpora gives us a new tool for tracking and ultimately understanding vaccine stance and sentiment.

## Appendix

Multimedia Appendix 1 Maternal vaccination keyword search.

Multimedia Appendix 2 Vaccination keyword search terms.

## Abbreviations

BERT: Bidirectional Encoder Representations from Transformers
GloVe: Global Vectors for Word Representation
LSTM: long short-term memory
NLP: natural language processing
TPU: Tensor Processing Unit
