# Supplementary material for: Categorizing Vaccine Confidence With a Transformer-Based Machine Learning Model: Analysis of Nuances of Vaccine Sentiment in Twitter Discourse
Source: JMIR Med Inform. 2021 Oct 8;9(10):e29584. doi: 10.2196/29584 (PMC8538052; doi:10.2196/29584)
Supplement: Multimedia Appendix 1 [file medinform_v9i10e29584_app1.docx]

### Appendix 1 - Maternal vaccination keyword search

**ENGLISH**

((("vaccin*" OR "immuniz*" OR "immunis*" OR "Tdap" OR ("vaccin*" NEAR/3 "pertussis") OR ("vaccin*" NEAR/3 "whooping cough") OR ("vaccin*" NEAR/3 "Tetanus") OR ("vaccin*" NEAR/3 "Influenza") OR ("vaccin*" NEAR/3 "flu") OR "flu shot*" OR "tetanus shot*" OR "whooping cough shot*" OR "pertussis shot*" OR ("vaccin*" NEAR/3 "Group B streptococcus") OR ("vaccin*" NEAR/3 "Respiratory Syncytial") OR ("vaccin*" NEAR/3 "GBS") OR ("vaccine*" NEAR/3 "RSV")) NEAR/8 (matern* OR pregna* OR antenatal) AND ("during pregna*" OR "while pregna*" OR "whilst pregna*" OR "when pregna*" OR "in pregna*" OR "are pregna*" OR "pregnant wom*")) OR "maternal immuniz*" OR "maternal Vaccin*" OR "maternal immunis*") NOT ("a vet" or veterinary OR dog* OR cat* OR horse* OR mouse* OR pig* OR cow* OR (financ* near/3 stock*) OR "immunoglobulin*" OR "LON:" OR "NYSE:" OR url:www.clinicaltrials.gov OR ((child* NEAR/1 vaccin*) AND (child* NEAR/1 vaccin*) AND (child* NEAR/1 vaccin*)))

**PORTUGUESE (with Brazilian text included)**

((("vacin*" OR "imuniz*" OR ("Tdap") OR ("vacin*" NEAR/3 "coqueluche") OR ("vacin*" NEAR/3 "tosse convulsa") OR ("vacin*" NEAR/3 "tétano") OR ("vacin*" NEAR/3 "influenza") OR ("vacin*" NEAR/3 "gripe") OR " injeção gripe *" OR " injeção tétano *" OR " injeção tosse convulsa *" OR " injeção coqueluche *" OR ("vacin*" NEAR/3 "estreptococo do grupo B") OR ("vacin*" NEAR/3 "sincicial respiratório") OR ("vacin*" NEAR/3 "SGB") OR ("vacin*" NEAR/3 "VSR")) NEAR/8 (matern* OR gravid*) AND ("durante gravidez*" OR "enquanto grávida*" OR "quando gravida*" OR "na gravidez*" OR "estão grávidas*" OR "mulher* grávida*")) OR "imuniz* matern*" OR "vacinação materna*" OR " imunização* matern*") NOT ("um veterinário" or veterinário OR cão* OR canídeo* OR gato* OR felino* OR cavalo* OR equino* OR rato* OR porco* OR suíno OR vaca* OR bovino OR (financ* near/3 gado*) OR “abastecimento” OR " imunoglobulina*" OR "LON:" OR "NYSE:" OR url:www.clinicaltrials.gov OR ((criança* NEAR/1 vacin*) AND (criança* NEAR/1 vacin*) AND (criança* NEAR/1 vacin*)))

**GERMAN**

((("vakzin*" OR "immunis*" OR "impf*" OR ("Tdap") OR ("impf*" NEAR/3 "Pertussis") OR ("impf*" NEAR/3 "Keuchhusten") OR ("impf*" NEAR/3 "Tetanus") OR ("impf*" NEAR/3 "Influenza") OR ("impf*" NEAR/3 "Grippe") OR "Grippe-Impfung*" OR "Grippeimpfung*" OR "Influenza-Impfung*" OR "Influenzaimpfung*" OR "Tetanus-Impfung*" OR "Tetanusimpfung*" OR "Keuchhusten-Impfung*" OR "Keuchhustenimpfung*" OR "Pertussis-Impfung*" OR "Pertussisimpfung*" OR ("impf*" NEAR/3 "Streptokokken Gruppe B") OR ("impf*" NEAR/3 "B-Streptokokken") OR ("impf*" NEAR/3 "Respiratorische Synzytial") OR ("impf*" NEAR/3 "GBS") OR ("impf*" NEAR/3 "RSV")) NEAR/8 (matern* OR schwanger*) AND ("während schwanger*" OR "solange schwanger*" OR "sobald schwanger*" OR "als schwanger*" OR "in der schwanger*" OR "sind schwanger*" OR "schwangere frau*")) OR "maternale Impf*" OR "maternale Immunis*" OR "maternale Vakzin*") NOT ("ein Tierarzt" OR Tierarzt OR Hund* OR Katze* OR Pferd* OR Maus* OR Schwein* OR Kuh* OR Kühe OR (finanz* near/3 Aktien*) OR "Immunoglobulin*" OR "LON:" OR "NYSE:" OR url:www.clinicaltrials.gov OR ((Kind* NEAR/1 impf*) AND (Kind* NEAR/1 impf*) AND (Kind* NEAR/1 impf*)))

**AFRIKAANS**

((entstof* OR ent OR inenting OR immunisering OR immuniseer OR (entstof* NEAR/3 pertussis) OR (entstof* NEAR/3 kinkhoes) OR (entstof* NEAR/3 tetanus) OR (entstof* NEAR/3 griep) OR griepinspuiting OR “griep spuit*” OR “griep spuite” OR “tetanus-inenting*” OR (entstof* NEAR/3 Groep B streptokokke) OR (entstof* NEAR/3 Respiratoriese sincytiale virus) OR (entstof* NEAR/3 GBS) OR (entstof* NEAR/3 RSV)) NEAR/8 (moeder* OR swanger*) AND (“terwyl swanger*” OR “tans swanger*” OR “wanneer swanger*” OR in swangerskap OR “is swanger*” OR “swanger vrou*” OR “verwagtende vrouens” OR “swanger vrouens” OR “swanger vroue”)) OR (moederinenting* OR “moeder inenting*” OR “moeder immunisering*”) NOT (“n veearts” OR veeartsenykundige OR hond* OR kat* OR perd* OR muis* OR vark* OR koei* OR (finansies* near/3 aandele*) OR immunoglobulien* OR url:www.clinicaltrials.gov OR ((kind* NEAR/1 entstof*) AND (kind* NEAR/1 entstof*) AND (kind* NEAR/1 entstof*)))

**TRADITIONAL CHINESE**

((("疫苗" OR "免疫" OR "免疫學" OR ("百白破") OR ("疫苗" NEAR/3 "百日咳") OR ("疫苗" NEAR/3 "破傷風") OR ("疫苗" NEAR/3 "流感") OR ("疫苗" NEAR/3 "感冒") OR "流感疫苗" OR "破傷風疫苗" OR "百白破疫苗" OR "白百破疫苗" OR ("疫苗" NEAR/3 " B組鏈球菌") OR ("疫苗" NEAR/3 "呼吸道合胞") OR ("疫苗" NEAR/3 "GBS") OR ("疫苗" NEAR/3 "RSV")) NEAR/8 (母 OR 孕產 OR產前) AND ("孕中" OR "孕期" OR "懷孕時" OR "當懷孕時" OR "懷孕中" OR "懷孕期間" OR "孕婦")) OR "產婦免疫接種" OR "孕期免疫" OR " 孕產婦免疫") NOT ("獸醫" or 動物醫生 OR 狗 OR 貓 OR 馬 OR 鼠 OR 豬 OR 牛 OR (金融 near/3 股票) OR "免疫球蛋白" OR "倫敦:" OR "紐約證券交易所:" OR url:www.clinicaltri als.gov OR ((兒童 NEAR/1 疫苗) AND (兒童 NEAR/1 免疫) AND (兒童 NEAR/1 免疫接種)))

**ITALIAN**

("vaccin*" OR "immuniz*" OR ("vaccin*" NEAR/3 "Tdap") OR ("vaccin" NEAR/3 "pertosse") OR ("vaccin*" NEAR/3 "tosse convulsiva") OR ("vaccin*" NEAR/3 "tosse asinina") OR ("vaccin*" NEAR/3 "tetano") OR ("vaccin*" NEAR/3 "influenz*") OR ("vaccin*" NEAR/3 "influenz*") OR "vaccinazione antinfluenzale" OR "vaccino antinfluenzale" OR "vaccinazione antitetanica" OR "vaccin* antitetanic*" OR " vaccino anti-pertosse " OR "vaccin* anti-tosse convulsiv*" OR "vaccin* antidifterite-tetano-pertosse" OR ("vaccin*" NEAR/3 "streptococco Gruppo B") OR ("vaccin*" NEAR/3 "virus repiratorio sinciziale") OR (“vaccin*” NEAR/3 "GBS") OR ("vaccin*" NEAR/3 "RSV")) NEAR/8 (matern* OR gravid*) AND ("durante la gravidanza" OR "mentre gravidanza" OR "in gravidanza" OR "incinta" OR "sono gravid*" OR "é gravid*" OR "donne gravide" OR "donna gravida" OR "donn* in gravidanza" OR "immunizzazione materna" OR "immunitá materna" OR "vaccinazione materna*") NOT (vet* or veterinar* OR can* OR gatt* OR cavall* OR top* OR maial* OR (finanz* NEAR/3 bestiame) OR immunoglobulin* OR ((bambin* NEAR/1 vaccin*) AND (bambin* NEAR/1 vaccin*) AND (bambin* NEAR/1 vaccin*)))

**SPANISH - SPAIN**

((("vacuna*" OR "inmuniz*" OR ("TDaP") OR ("vacuna" NEAR/3 "DPT") OR ("vacuna" NEAR/3 "difteria-tétanos-pertussis") OR ("vacuna" NEAR/3 "DTPa") OR ("vacuna" NEAR/3 "DT") OR ("vacuna" NEAR/3 "triple bacteriana") OR ("vacuna" NEAR/3 "pertussis") OR ("vacuna" NEAR/3 "tos convuls*") OR ("vacuna" NEAR/3 "tos ferina") OR ("vacuna" NEAR/3 "coqueluche") OR ("vacuna" NEAR/3 "tétanos") OR ("vacuna" NEAR/3 "tétano") OR ("vacuna" NEAR/3 "difteria") OR ("vacuna" NEAR/3 "Influenza") OR ("vacuna" NEAR/3 "gripe") OR "vacuna antigripal" OR "vacuna antiinfluenza" OR “vacuna antitetánica” OR "toxoide tetánico" OR "vacuna antipertussis" OR "vacuna antipertusis" OR "vacuna antipertúsica" OR “vacuna anticoqueluchosa” OR "vacuna antidiftérica" OR “toxoide diftérico” OR ("vacuna" NEAR/3 "streptococcus del grupo B") OR ("vacuna" NEAR/3 "estreptococos del grupo B") OR ("vacuna" NEAR/3 "respiratorio sincicial") OR ("vacuna" NEAR/3 "sincicial respiratorio") OR ("vacuna" NEAR/3 "GBS") OR ("vacuna" NEAR/3 "EGB") OR ("vacuna" NEAR/3 "SGB") OR ("vacuna" NEAR/3 "RSV") OR ("vacuna" NEAR/3 "VRS"))

NEAR/8 (matern* OR embaraz* OR gestación OR preñez OR madres) AND ("durante el embarazo" OR "mientras embarazadas" OR "cuando están embarazadas" OR "durante la preñez" OR "durante la gestación" OR "en el embarazo" OR "en gestantes" OR "estas embarazada" OR "muje* embaraz*" OR "muje* gestantes" OR “la gestante”)) OR "inmunización matern*" OR "vacuna* matern*") NOT (veterinari* OR perr* OR canin* OR gat* OR felin* OR equin* OR caballo* OR yegu* OR rat* OR roedo* OR cerd* OR porcin* OR marrana* OR vacas OR bovin* OR vacuno OR aves OR aviar OR (financia* NEAR/3 valores) OR "abastecimiento" OR "immunoglobulin*" OR "LON:" OR "NYSE:" OR url:www.clinicaltrials.gov OR ((niñ* NEAR/1 vacuna*) AND (niñ* NEAR/1 vacuna*) AND (niñ* NEAR/1 vacuna*)))

**SPANISH – MEXICO**

((("vacuna*" OR "inmuniz*" OR ("TDaP") OR ("vacuna" NEAR/3 "DPT") OR ("vacuna" NEAR/3 "difteria-tétanos-pertussis") OR ("vacuna" NEAR/3 "difteria-tétanos-tos ferina") OR ("vacuna" NEAR/3 "DTPa") OR ("vacuna" NEAR/3 "DT") OR ("vacuna" NEAR/3 "triple bacteriana") OR ("vacuna" NEAR/3 "pertussis") OR ("vacuna" NEAR/3 "tos convuls*") OR ("vacuna" NEAR/3 "tos ferina") OR ("vacuna" NEAR/3 "coqueluche") OR ("vacuna" NEAR/3 "tétanos") OR ("vacuna" NEAR/3 "tétano") OR ("vacuna" NEAR/3 "difteria") OR ("vacuna" NEAR/3 "influenza") OR ("vacuna" NEAR/3 "gripe") OR ("vacuna" NEAR/3 "gripa") OR "vacuna antigripal" OR "vacuna antiinfluenza" OR “vacuna antitetánica” OR "toxoide tetánico" OR "vacuna antipertussis" OR "vacuna antipertusis" OR "vacuna antipertúsica" OR “vacuna anticoqueluchosa” OR "vacuna antidiftérica" OR “toxoide diftérico” OR ("vacuna" NEAR/3 "streptococcus del grupo B") OR ("vacuna" NEAR/3 "estreptococos del grupo B") OR ("vacuna" NEAR/3 "respiratorio sincicial") OR ("vacuna" NEAR/3 "sincicial respiratório") OR ("vacuna" NEAR/3 "GBS") OR ("vacuna" NEAR/3 "EGB") OR ("vacuna" NEAR/3 "SGB") OR ("vacuna" NEAR/3 "RSV") OR ("vacuna" NEAR/3 "VRS")) NEAR/8 (matern* OR embaraz* OR gestación OR gravidez OR madres) AND ("durante el embarazo" OR "mientras embarazadas" OR "cuando están embarazadas" OR "durante la gravidez" OR "durante la gestación" OR "en el embarazo" OR "en gestantes" OR "estas embarazada" OR "muje* embaraz*" OR "muje* gestantes" OR “la gestante” OR “durante la gravidez”)) OR "inmunización matern*" OR "vacuna* matern*") NOT (veterinari* OR perr* OR canin* OR gat* OR felin* OR equin* OR caballo* OR yegu* OR rat* OR roedo* OR cerd* OR porcin* OR marrana* OR vacas OR bovin* OR vacuno OR aves OR pájaros OR aviar OR (financia* NEAR/3 valores) OR "abastecimiento" OR "immunoglobulin*" OR "LON:" OR "NYSE:" OR url:www.clinicaltrials.gov OR ((niñ* NEAR/1 vacuna*) AND (niñ* NEAR/1 vacuna*) AND (niñ* NEAR/1 vacuna*)))

**SPANISH – PANAMA**

((("vacuna*" OR "inmuniz*" OR "TDaP" OR “DPT” OR “DTP” OR (“vacuna contra difteria tétanos y tosferina”) OR ("vacuna" NEAR/3 "pertussis") OR (“tosferina” OR “coqueluche” OR “tos convuls*”)) OR ("vacuna" NEAR/3 "tosferina” OR (“tos convuls*" OR “coqueluche”)) OR ("vacuna" NEAR/3 "tétan*") OR ("vacuna" NEAR/3 "influenza") OR (“resfriado*” OR “catarro*”) OR ("vacuna" NEAR/3 "gripe") OR (“resfriad*” OR “catarro*” OR “influenza”)) OR “vacuna antigripal” OR “vacuna antiinfluenza” OR “vacuna antitetánica” OR (“toxoide antitetánico”) OR “vacuna contra la tosferina” OR ("vacuna contra pertussis ") OR ("vacuna" NEAR/3 "estreptococos del grupo B" OR “EGB”) OR ("vacuna" NEAR/3 "respiratorio sincicial") OR (“sincicial respiratorio”) OR ("vacuna" NEAR/3 "SGB") OR ("vacuna" NEAR/3 "VRS")) NEAR/8 (matern* OR embaraz* OR gestación OR preñez OR prenatal) AND (“durante el embarazo” OR “mientras el embarazo” OR “cuando están embarazadas” OR “durante la preñez” OR “durante la gestación” OR “en el embarazo” OR “en gestantes” OR “estas embarazada” OR “muje* embaraz*” OR “muje* gestantes” OR “la gestante”) OR (“inmunización matern*” OR “vacuna* matern*”) NOT (veterinari* OR perr* OR canin* OR gat* OR felin* OR caball* OR equin* OR rat* OR roedo* OR cerd* OR porcin* OR marran* OR vaca* OR bovin* OR vacuno OR (financ* OR finanz* NEAR/3 valores) OR abastecimiento OR surtid* OR inventario* OR suministro* OR existencia* OR immunoglobulin* OR (((niñ* OR chic* OR pequeñ* OR crí* OR infant* OR menor*) NEAR/1 vacuna*) AND ((niñ* OR chic* OR pequeñ* OR crí* OR infant* OR menor*) NEAR/1 vacuna*) AND ((niñ* OR chic* OR pequeñ* OR crí* OR infant* OR menor*) NEAR/1 vacuna*)))

**FRENCH**

((("vaccin*" OR "immunis*" OR "dTP" OR ("vaccin*" NEAR/3 "coqueluche") OR ("vaccin*" NEAR/3 "dTPca") OR ("vaccin*" NEAR/3 "tétanos") OR ("vaccin*" NEAR/3 "grippe") OR ("vaccin*" NEAR/3 "antigrippal") OR "vaccin contre la grippe*" OR "vaccin contre le tétanos*" OR "vaccin contre la coqueluche*" OR ("vaccin*" NEAR/3 "streptocoque du Groupe B") OR ("vaccin*" NEAR/3 "virus respiratoire syncytial") OR ("vaccin*" NEAR/3 "SGB") OR ("vaccin*" NEAR/3 "VRS")) NEAR/8 (enceinte* OR grossesse*) AND ("pendant la grossesse" OR "lors de la grossesse" OR "chez les femmes enceintes" OR "chez la femme enceinte")) OR "vaccination maternelle") NOT (vétérinaire OR chien* OR chat* OR cheva* OR souris* OR cochon* OR vache* OR (financ* near/3 stock*) OR "immunoglobuline*" OR "LON:" OR "NYSE:" OR url:www.clinicaltrials.gov OR ((enfant* NEAR/3 vaccin*) AND (enfant* NEAR/3 vaccin*) AND (enfant* NEAR/3 vaccin*)))

**FRENCH - CANADA**

((("vaccin*" OR "immunis*" OR ("dTP") OR ("vaccin*" NEAR/3 "coqueluche") OR ("vaccin*" NEAR/3 "dTPca") OR ("vaccin*" NEAR/3 "Tetanos") OR ("vaccin*" NEAR/3 "grippe") OR ("vaccin*" NEAR/3 "antigrippal") OR ("vaccin*" NEAR/3 "streptocoque B") OR ("vaccin*" NEAR/3 "SGB") OR ("vaccin*" NEAR/3 "virus respiratoire syncytial") OR ("vaccin*" NEAR/3 " streptocoque du Groupe B") OR ("vaccin*" NEAR/3 "VRS")) NEAR/8 (enceinte* OR grossesse*) AND ("pendant la grossesse" OR "lors de la grossesse" OR "chez les femmes enceintes" OR "chez la femme enceinte")) OR "vaccination maternelle") NOT (vétérinaire OR chien* OR chat* OR cheva* OR souris* OR cochon* OR vache* OR (financ* near/3 stock*) OR "immunoglobuline*" OR "LON:" OR "NYSE:" OR url:www.clinicaltrials.gov OR ((enfant* NEAR/3 vaccin*) AND (enfant* NEAR/3 vaccin*) AND (enfant* NEAR/3 vaccin*)))

**ZULU**

((("ukugoma" OR "Umgomo" OR " Ukugonywa” OR “ Ukugoma” OR ("Tdap") OR TT OR Td OR ("ukugoma*" NEAR/3 "pertussis") OR ("ukugoma*" NEAR/3 " Ukukhwehlela ") OR ("ukugoma*" NEAR/3 "Tetanus") OR ("ukugoma*" NEAR/3 " Umkhuhlane") OR ("ukugoma*" NEAR/3 " Umkhuhlane") OR "umjovo womkhuhlane” OR "tetanus womkhuhlane*" OR "whooping cough womkhuhlane*" OR "pertussis womkhuhlane*" OR ("ukugoma*" NEAR/3 "Group B streptococcus") OR ("ukugom*" NEAR/3 "Respiratory Syncytial") OR ("ukugoma*" NEAR/3 "GBS") OR ("ukugomo*" NEAR/3 "RSV")) NEAR/8 (matern* OR pregna*) AND ("during pregna*" OR "ngenkathi ekhulelwe" OR " uma ekhulelwe" OR " uma ukhulelwe" OR "ekukhulelweni" OR "are pregna*" OR " owesifazane okhulelwe")) OR " ukugoma komama" OR "ukugoma komama" OR " ukugoma komama*")

NOT ("udokotela wezilwane" or “udokotela wezilwane” OR inja OR ikati OR Ihhashi OR igundane OR ingulube OR inkomo OR (Izimali near/3 isitoko) OR " izakhamzimba*" OR "LON:" OR "NYSE:" OR url:www.clinicaltrials.gov OR (((umntwana OR ebunganeni OR izingane) NEAR/1 Umgo) AND ((umntwana OR ebunganeni OR izingane) NEAR/1 Umgo) AND ((umntwana OR ebunganeni OR izingane) NEAR/1 Umgo)))

**KOREAN**

((("백신*" OR "면역*" OR "면역*" OR "Tdap" OR ("백신*" NEAR/3 "백일해") OR ("백신*" NEAR/3 "백일해") OR ("백신*" NEAR/3 "파상풍") OR ("백신*" NEAR/3 "독감") OR ("백신*" NEAR/3 "독감") OR "독감 주사*" OR "파상풍 주사*" OR "백일해 주사*" OR "백일해 주사*" OR ("백신*" NEAR/3 ("B그룹 연쇄상구균" OR “그룹 B 연쇄상구균”)) OR ("백신*" NEAR/3 "공기 세포융합") OR ("백신*" NEAR/3 " B그룹 연쇄상구균") OR ("백신*" NEAR/3 "공기 세포융합 바이러스"))

NEAR/8 (모체* OR 임신* OR 출산) AND ("임부*" OR "임신 중*" OR "임신했*" OR "임신*" OR "임신한*" OR "임신한*" OR "임산부*")) OR "모체 면역*" OR "모체이행면역*" OR "모자간 면역*" OR "모성면역*" OR "모아면역*" OR "모체이행항체*")

NOT ("수의사" 가축 OR 동물 OR 개* OR 고양이* OR 말* OR 쥐* OR 돼지* OR 소* OR ((금융* OR 재무* OR 재정*) near/3 가축*) OR "면역 글로불린*" OR "LON:" OR "NYSE:" OR url:www.clinicaltrials.gov OR ((어린이* NEAR/1 백신*) AND (유아* NEAR/1 백신*) AND (아기* NEAR/1 백신*)))

**HINDI**

(“वैक्सीन” OR "टीका*" OR “टीका लगाना” OR “वैक्सीन” "प्रतिरक्षण" OR "रोगक्षम करना" OR "Tdap" OR "TT" OR "Td" OR "DT" OR ("वैक्सीन" NEAR/3 "पर्टुसिस") OR ("वैक्सीन" NEAR/3 "इंफ्लुएंजा") OR ("वैक्सीन" NEAR/3 "फ्लू") OR ("वैक्सीन" NEAR/3 "टिटेनस") OR “टी टी” OR "फ्लू का टीका*" OR "टीटी*" OR "काली खांसी का टीका" OR "पर्टुसिस का टीका*" OR ("टीका*" NEAR/3 "ग्रूप B स्ट्रेप्टोकोक्कस") OR ("टीका*" NEAR/3 "श्वसन संबंधी") OR ("टीका*" NEAR/3 "गिओन बार") OR ("टीका*" NEAR/3 "आरएसवी")) NEAR/8 ("गर्भवती" OR "गर्भिणी" OR "गर्भवती होने"OR "जबकि गर्भवती" OR "गर्भावस्था में" OR "गर्भवती हैं" OR "गर्भवती महिला" OR "गर्भवती महिलाओं" OR "मातृ टीकाकरण") NOT ("पशु चिकित्सक" or “पशुचिकित्सा” OR कुत्ता OR बिल्ली OR घोड़ा OR चूहा O सूअर OR गाय OR "LON:" OR "NYSE:" OR url:www.clinicaltrials.gov OR ((बच्चा NEAR/1 वैक्सीन) AND (बच्चा NEAR/1 वैक्सीन) AND (बच्चा NEAR/1 वैक्सीन)) OR ((शिशु NEAR/1 वैक्सीन) AND (शिशु NEAR/1 वैक्सीन) AND (शिशु NEAR/1 वैक्सीन)) OR ((बच्चे NEAR/1 वैक्सीन) AND (बच्चा NEAR/1 टीके) AND (बच्चा NEAR/1 टीका)) OR ((बच्चे NEAR/1 वैक्सीन) AND (बच्चे NEAR/1 टीका) AND (बच्चे NEAR/1 वैक्सीन)) OR ((बच्चे NEAR/1 टीके) AND (बचपन NEAR/1 वैक्सीन) AND (बचपन NEAR/1 टीका)) OR ((बचपन NEAR/1 टीके) AND (बचपन NEAR/1 वैक्सीन) AND (बचपन NEAR/1 वैक्सीन)))

**BENGALI**

((("টিকা*" OR "ভ্যাকসি*" OR "টীকা*" OR "টিডাপ" OR ("টিকা*" NEAR/3 "পার্টুসিস") OR ("টিকা*" NEAR/3 "হুপিং কাশি") OR ("টিকা*" NEAR/3 "টিটেনাস") OR ("টিকা*" NEAR/3 "ইনফ্লুয়েঞ্জা") OR ("টিকা*" NEAR/3 "ফ্লু") OR "ফ্লু শট*" OR "টিটেনাস ইনজেকশন*" OR "হুপিং কাশির ইনজেকশন*" OR "পার্টুসিস শট*" OR ("টিকা*" NEAR/3 "গ্রুপ বি স্ট্রেপ্টোকক্কাস") OR ("টিকা*" NEAR/3 "রেসপিরেটরি সিনসাইশিয়াল") OR ("টিকা*" NEAR/3 "জিবিএস") OR ("টিকা*" NEAR/3 "আরএসভি"))

NEAR/8 (প্রস* OR গর্ভবতী* OR প্রসবের পর) AND ("গর্ভবতী অবস্থায়*" OR "প্রেগনেন্ট অবস্থায়*" OR "প্রেগন্যান্ট থাকার সময়*" OR "গর্ভবতী থাকা*" OR "গর্ভাবস্থায়*" OR "গর্ভবতী*" OR "গর্ভবতী মহিলা*")) OR "মায়ের টিকা*" OR "মায়েদের টিকা*" OR "মায়ের জন্য টিকা*") NOT ("পশুচিকিৎসক" or ভেটেরেনারি OR কুকুর* OR বিড়াল* OR ঘোড়া* OR ইঁদুর* OR শুকর* OR গরু* OR (আর্থিক* near/3 স্টক*) OR "ইমিউনোগ্লোবিউলিন*" OR "LON:" OR "NYSE:" OR url:www.clinicaltrials.gov OR ((শিশু* NEAR/1 টিকা*) AND (শিশু* NEAR/1 টিকা*) AND (শিশু* NEAR/1 টিকা*)))

**TELUGU**

((("’టీకా*" OR "వ్యాధినిరోధక*" OR "వ్యాధినిరోధక*" OR "టిడాప్" OR ("టీకా*" NEAR/3 "కోరింతదగ్గు") OR ("టీకా*" NEAR/3 "కోరింత దగ్గు") OR ("టీకా*" NEAR/3 "ధనుర్వాతం") OR ("టీకా*" NEAR/3 "ఇన్‌ఫ్లూయెంజా") OR ("టీకా*" NEAR/3 "ఫ్లూ జ్వరం") OR "ఫ్లూ జ్వరం ఇంజెక్షన్*" OR "ధనుర్వాతం ఇంజెక్షన్*" OR "కోరింత దగ్గు ఇంజెక్షన్*" OR " కోరింతదగ్గు ఇంజెక్షన్ *" OR ("టీకా*" NEAR/3 "గ్రూప్ బి స్ట్రెప్టోకాకస్") OR ("టీకా*" NEAR/3 "శ్వాస సంబంధ సిన్సిటియల్") OR ("టీకా*" NEAR/3 "గులియన్ బేర్ సిండ్రోం") OR ("టీకా*" NEAR/3 "శ్వాస సంబంధ సిన్సిటియల్ వైరస్"))

NEAR/8 (ప్రసూతి* OR గర్భం* OR గర్భస్థ) AND ("గర్భధారణ సమయంలో*" OR "గర్భవతిగా ఉండగా*" OR "గర్భవతి అయినప్పుడు*" OR "గర్భం ధరించినప్పుడు*" OR "గర్భధారణలో*" OR "గర్భం ధరించివున్నారు*" OR "గర్భిణీ స్త్రీ*")) OR "తల్లికి టీకా*" OR "తల్లికి టీకా*" OR "తల్లికి వ్యాధినిరోధక*")

NOT ("ఒక పశు" లేదా పశుసంబంధ OR కుక్క* OR పిల్లి* OR గుర్రం* OR ఎలుక* OR పంది* OR ఆవు* OR (ఫినాన్స్* near/3 పశుగణ*) OR "ఇమ్యునోగ్లోబిన్*" OR "LON:" OR "NYSE:" OR url:www.clinicaltrials.gov OR ((బిడ్డ* NEAR/1 టీకా*) AND (బిడ్డ* NEAR/1 టీకా*) AND (బిడ్డ* NEAR/1 టీకా *)))

**TAMIL**

((("தடுப்பூசி*" OR "நோயெதிர்ப்பு*" OR "நோயெதிர்ப்புத் திறனூட்டுதல்*" OR "முத்தடுப்பு ஊசி" OR ("தடுப்பூசி*" NEAR/3 "கக்குவான்") OR ("தடுப்பூசி*" NEAR/3 " கக்குவான் இருமல்") OR ("தடுப்பூசி*" NEAR/3 "டெட்டனஸ்") OR ("தடுப்பூசி*" NEAR/3 "சளிக்காய்ச்சல்") OR ("தடுப்பூசி*" NEAR/3 "ஃப்ளூ") OR "ஃப்ளூ ஊசி*" OR "டெட்டனஸ் ஊசி*" OR "கக்குவான் இருமல் ஊசி*" OR "கக்குவான் ஊசி*" OR ("தடுப்பூசி*" NEAR/3 "குழு B ஸ்ட்ரெப்டோகாக்கஸ்") OR ("தடுப்பூசி*" NEAR/3 "மூச்சக இணைச்செல்லிய வைரஸ்") OR ("தடுப்பூசி*" NEAR/3 " GBS") OR ("தடுப்பூசி*" NEAR/3 "RSV")) NEAR/8 (மகப்பேறு* OR கர்ப்பம்* OR பேறுகாலத்திற்கு முன்) AND ("கர்ப்பத்தின் போது*" OR "கர்ப்பமாக இருக்கும்போது*" OR "கர்ப்பமாக இருக்கும் நேரத்தில்*" OR "கர்ப்பம் அடைந்திருக்கும்போது*" OR "கர்ப்பத்தில்*" OR "கர்ப்பமாக இருக்கிறார்கள்*" OR "கர்ப்பிணி பெண்*")) OR "மகப்பேறு நோயெதிர்ப்பு*" OR "மகப்பேறு தடுப்பூசி*" OR "மகப்பேறு நோயெதிர்ப்புத் திறனூட்டுதல்*") NOT ("ஒரு கால்நடை மருத்துவர்" OR கால்நடை மருத்துவம் OR நாய்* OR பூனை* OR குதிரை* OR எலி* OR பன்றி* OR பசு* OR (நிதி* near/3 இருப்பு*) OR "நோய் எதிர்ப்புப் புரதம்*" OR "LON:" OR "NYSE:" OR url:www.clinicaltrials.gov OR ((குழந்தை* NEAR/1 தடுப்பூசி*) AND (குழந்தை* NEAR/1 தடுப்பூசி*) AND (குழந்தை* NEAR/1 தடுப்பூசி*)))

**MARATHI**

((("लस*" OR "लसीकरण*" OR " लस देणे*" OR "Tdap" OR ("लस*" NEAR/3 "डांग्या खोकला") OR ("लस*" NEAR/3 " व्हूपिंग खोकला") OR ("लस*" NEAR/3 "धनुर्वात") OR ("लस *"NEAR/3 "शीतज्वर") OR ("लस*" NEAR/3 "फ्ल्यू") OR "फ्ल्यूची लस*" OR "धनुर्वात लस*" OR " व्हूपिंग खोकल्याची लस*" OR " डांग्या खोकल्याची लस*" OR ("लस *" NEAR/3 "समूह B स्ट्रेप्टोकॉकस ") OR ("लस *" NEAR/3 " श्वसनविषयक संश्लेषण ") OR ("लस *" NEAR/3 "स्नायुंचा अशक्तपणा” OR “जीबीएस") OR ("लस *" NEAR/3 "संपेशिका” OR “आरएसव्ही")) NEAR/8 (प्रसूती* OR गर्भावस्था* OR प्रसूतिपूर्व) AND ("गर्भावस्थेदरम्यान*" OR "गर्भावस्थेत असताना*" OR "गर्भावस्था असताना*" OR "जेव्हा गर्भावस्था असते*" OR "गर्भावास्थेमध्ये*" OR "गर्भवती आहेत*" OR "गर्भवती महिला*")) OR "गर्भावस्थेतील लसीकरण*" OR "गर्भावस्थेतील लस*" OR "गर्भावस्थेत लस देणे*") NOT ("एक पशुचिकित्सक" or पशुचिकित्सकीय OR कुत्रा* OR मांजर* OR घोडा* OR उंदीर* OR डुक्कर* OR गाय* OR (अर्थसहाय्य* near/3 साठा*) OR "इम्युनोग्लोब्युलिन*" OR "LON:" OR "NYSE:" OR url:www.clinicaltrials.gov OR ((मूल* NEAR/1 लस*) AND (बाळ* NEAR/1 लस*) AND (अर्भक* NEAR/1 लस*)))

**URDU**

((("ویکسین*" OR "مامون*" OR "ڈی ٹی پی" OR ("ویکسین*" NEAR/3 "شہقہ") OR ("ویکسین *" NEAR/3 "کالی کھانسی") OR ("ویکسین *" NEAR/3 "ٹیٹنس") OR ("ویکسین *" NEAR/3 "انفلوئنزا") OR ("ویکسین *" NEAR/3 "زکام") OR "انفلوئنزا کا ٹیکہ*" OR "ٹیٹنس کا ٹیکہ*" OR "کالی کھانسی کا ٹیکہ*" OR "شہقہ کا ٹیکہ*" OR ("ویکسین *" NEAR/3 "سٹریپٹو کو کس گروپ بی") OR ("ویکسین *" NEAR/3 "تنفسی کثیر نواتی") OR ("ویکسین *" NEAR/3 "جی بی ایس") OR ("ویکسین *" NEAR/3 "RSV")) NEAR/8 (مادرانہ* OR حمل* OR وضع حمل) AND ("دوران حمل*" OR "حمل میں*" OR "حمل کے دوران*" OR "جب حاملہ*" OR "حمل سے*" OR "حاملہ*" OR "حاملہ عورت*")) OR "مادرانہ مامونیت*" OR "مادرانہ ویکسین *" OR "مادرانہ مامونیت*") NOT ("ایک بیطاری" یا مویشیوں کا معالج OR کتا* OR بلی* OR گھوڑا* OR چوہا* OR سور* OR گائے* OR (سرمایا* near/3 حصص*) OR "ضد جسم*" OR "LON:" OR "NYSE:" OR url:www.clinicaltrials.gov OR ((بچہ* NEAR/1 ویکسین*) AND (بچہ* NEAR/1 ویکسین*) AND (بچہ* NEAR/1 ویکسین*)))

###

### 
