# Supplementary material for: Categorizing Vaccine Confidence With a Transformer-Based Machine Learning Model: Analysis of Nuances of Vaccine Sentiment in Twitter Discourse
Source: JMIR Med Inform. 2021 Oct 8;9(10):e29584. doi: 10.2196/29584 (PMC8538052; doi:10.2196/29584)
Supplement: Multimedia Appendix 2 [file medinform_v9i10e29584_app2.docx]

### Appendix 2 - Vaccination keyword search terms

"vaccin*" OR “vax” OR “vaxer” or “vaxers” OR “vaxx” OR “vaxxer” OR “vaxxers” OR "immuniz*" OR "immunis*" OR (("shot" OR “jab”) NEAR/4 ("influenza" OR “influensa”)) OR (("shot" OR “jab”) NEAR/4 "flu") OR (("shot" OR “jab”) NEAR/4 "virus") OR (("shot" OR “jab”) NEAR/4 "whooping") OR (("shot" OR “jab”) NEAR/4 "pertussis") OR (("shot" OR “jab”) NEAR/4 "strepto*") OR (("shot" OR “jab”) NEAR/4 "respiratory") OR (("shot" OR “jab”) NEAR/4 "syncytial") OR (("shot" OR “jab”) NEAR/4 "rzv") OR (("shot" OR “jab”) NEAR/4 "rsv") OR (("shot" OR “jab”) NEAR/4 "h1n1") OR (("shot" OR “jab”) NEAR/4 "zvl") OR (("shot" OR “jab”) NEAR/4 "pcv13") OR (("shot" OR “jab”) NEAR/4 "ppsv23") OR (("shot" OR “jab”) NEAR/4 "menb") OR (("shot" OR “jab”) NEAR/4 "varicella") OR (("shot" OR “jab”) NEAR/4 "syncytial") OR (("shot" OR “jab”) NEAR/4 "tdap") OR (("shot" OR “jab”) NEAR/4 "dtap") OR

(("shot" OR “jab”) NEAR/4 "pertussis") OR (("shot" OR “jab”) NEAR/4 "tetanus") OR (("shot" OR “jab”) NEAR/4 "measles") OR (("shot" OR “jab”) NEAR/4 "cholera") OR (("shot" OR “jab”) NEAR/4 "rota*") OR (("shot" OR “jab”) NEAR/4 "ebola") OR (("shot" OR “jab”) NEAR/4 "hepat*") OR (("shot" OR “jab”) NEAR/4 "hepa") OR (("shot" OR “jab”) NEAR/4 "hepb") OR (("shot" OR “jab”) NEAR/4 "hib") OR

(("shot" OR “jab”) NEAR/4 "heamophilous") OR (("shot" OR “jab”) NEAR/4 "hpv") OR (("shot" OR “jab”) NEAR/4 "papilloma*") OR (("shot" OR “jab”) NEAR/4 "cervical") OR (("shot" OR “jab”) NEAR/4 "encephalitis") OR (("shot" OR “jab”) NEAR/4 "cholera") OR (("shot" OR “jab”) NEAR/4 "malaria") OR (("shot" OR “jab”) NEAR/4 "meningitis") OR (("shot" OR “jab”) NEAR/4 "polio*") OR (("shot" OR “jab”) NEAR/4 "pnemonia*") OR (("shot" OR “jab”) NEAR/4 "pneumonia*") OR (("shot" OR “jab”) NEAR/4 "mmr") OR (("shot" OR “jab”) NEAR/4 "varicella") OR (("shot" OR “jab”) NEAR/4 "var") OR (("shot" OR “jab”) NEAR/4 "chickenpox") OR (("shot" OR “jab”) NEAR/4 "chicken pox") OR (("shot" OR “jab”) NEAR/4 "zoster") OR (("shot" OR “jab”) NEAR/4 "yellow fever") OR (("shot" OR “jab”) NEAR/4 "zika") OR (("shot" OR “jab”) NEAR/4 "gbs") OR (("shot" OR “jab”) NEAR/4 "rubella*")
